# Anti-hyperuricemia effect of *Clerodendranthus spicatus*: a molecular biology study combined with metabolomics

**DOI:** 10.1038/s41598-024-66454-7

**Published:** 2024-07-04

**Authors:** Zheng Zhou, Manfei Xu, Meng Bian, Anzheng Nie, Bao Sun, Chunsheng Zhu

**Affiliations:** 1 Department of Chinese Medicine, The First Affiliated Hospital of Zhengzhou University, 1 Jianshe Road, Zhengzhou, 450000 China; 2 Department of Pharmacy, The Second Xiangya Hospital, Central South University, 139 Renmin Middle Road, Changsha, 410011 China; 3 Institute of Clinical Pharmacy, Central South University, Changsha, 410011 China

**Keywords:** *Clerodendranthus spicatus*, Hyperuricemia, Uric acid transporters, Metabolomics, Molecular biology, Nephrology

## Abstract

Hyperuricemia (HUA), a metabolic disease caused by excessive production or decreased excretion of uric acid (UA), has been reported to be closely associated with a variety of UA transporters. *Clerodendranthus spicatus* (*C. spicatus*) is an herbal widely used in China for the treatment of HUA. However, the mechanism has not been clarified. Here, the rat model of HUA was induced via 10% fructose. The levels of biochemical indicators, including UA, xanthine oxidase (XOD), adenosine deaminase (ADA), blood urea nitrogen (BUN), and creatinine (Cre), were measured. Western blotting was applied to explore its effect on renal UA transporters, such as urate transporter1 (URAT1), glucose transporter 9 (GLUT9), and ATP-binding cassette super-family G member 2 (ABCG2). Furthermore, the effect of *C. spicatus* on plasma metabolites was identified by metabolomics. Our results showed that *C. spicatus* could significantly reduce the serum levels of UA, XOD, ADA and Cre, and improve the renal pathological changes in HUA rats. Meanwhile, *C. spicatus* significantly inhibited the expression of URAT1 and GLUT9, while increased the expression of ABCG2 in a dose-dependent manner. Metabolomics showed that 13 components, including 1-Palmitoyl-2-Arachidonoyl-sn-glycero-3-PE, Tyr-Leu and N-cis-15-Tetracosenoyl-C18-sphingosine, were identified as potential biomarkers for the UA-lowering effect of *C. spicatus*. In addition, pathway enrichment analysis revealed that arginine biosynthesis, biosynthesis of amino acids, pyrimidine metabolism and other metabolic pathways might be involved in the protection of *C. spicatus* against HUA. This study is the first to explore the mechanism of anti-HUA of *C. spicatus* through molecular biology and metabolomics analysis, which provides new ideas for the treatment of HUA.

## Introduction

UA (UA), as the final product of endogenous sources from self-damaged and dead cells or exogenous sources from high-purine diet, is mainly synthesized in the liver^1^. It is well known that hyperuricemia (HUA), defined as abnormal excess accumulation of UA, is caused by the overproduction of UA or the reduced excretion of UA from the kidneys and intestines^2^. According to recent epidemiological data, the prevalence of HUA is gradually increasing^3^. Particularly, it has been shown that the prevalence of HUA is 13.3% (19.4% in men and 7.9% in women) in China^4^ and 21% (21.2% in men and 21.6% in women) in the United States^5^. Of note, evidence indicates that HUA is not only a predisposing factor for gout, but also a crucial risk factor for the development of other metabolic diseases^6^. For instance, a multicenter retrospective real-world study showed that HUA was associated with dyslipidemia and chronic kidney disease both cross-sectionally and longitudinally^7^. More recently, another longitudinal cohort study reported that HUA significantly increased the risk of cardiovascular disease and all-cause mortality^8^. Undoubtedly, HUA deserves further attention.

Considering that HUA is a major risk factor for the progress of many metabolic diseases, it is possible that appropriate control of UA contributes to the prevention and treatment of these diseases. Currently, there are two main mechanisms by which UA-lowering drugs work: (1) Drugs that inhibit UA synthesis, such as allopurinol and febuxostat, are often recommended as first-line clinical drugs^6^. Nevertheless, clinical data indicate that these drugs have a series of side effects or poor efficacy, including hypersensitivity syndrome, worsening renal function and cardiovascular disease death^9,10^; (2) Drugs that promote UA excretion, including benzbromarone, have been used clinically for 30 years^11^. Whereas, it has been withdrawn from the European market due to its severe hepatotoxicity^12^. Consequently, under this case, safer and more effective drugs are needed to prevent HUA and related diseases.

*Clerodendranthus spicatus (Thunb.)* C. Y. Wu (*C. spicatus*), commonly known as ‘kidney tea’ in China and ‘Kumis kucing’ in Southeast Asia, is a perennial herb from the *Lamiaceae* family^13,14^. In the Chinese folk medicine, *C. spicatus* is frequently administered as a functional tea to prevent and treat various diseases, such as urinary lithangiuria, rheumatism, and gout^13,15^. Of note, preclinical studies have found that *C. spicatus* significantly reduce UA and blood urea nitrogen (BUN) levels, and dramatically improved renal tubular injury in rats with HUA nephropathy^16^. Increasing evidence suggests that *C. spicatus* and its compounds have numerous biological activities, including anti-tumor, anti-HUA, anti-gouty arthritis, analgesia, and anti-hyperglycemia^13,17,18^. However, according to literature surveys, there are fewer reports on the anti-HUA mechanisms of *C. spicatus*. Molecular biology analysis and metabolomics are two main approaches for understanding biological processes, and their combination will bring new information to pharmacological studies^19,20^. Hence, we explored the effects of *C. spicatus* on UA transporters and plasma metabolic profiles in HUA rats. This study is expected to provide a theoretical basis for the mechanisms by which *C. spicatus* ameliorates HUA and present new ideas for the treatment of HUA.

## Materials and methods

### Chemicals and reagents

Acetonitrile and formic acid (UPLC grade) were obtained from Merk (Darmstadt, Germany) and Sigma Aldrich (St. Louis, USA), respectively. Ultrapure water was obtained from Millipore-Q water purification system (Millipore, USA). All the other chemicals were purchased through market resources.

Sample of *C. spicatus* was purchased from Xishuangbanna (Yunnan Province, China) and authenticated by Professor Binghong Fei (Department of Traditional Chinese Medicine, The First Affiliated Hospital of Zhengzhou University, Zhengzhou, China). *C. spicatus* was crushed into powder, and 1500 g of the powder was accurately weighed and extracted with 1:10 of water by heating to reflux for 1 h. Such procedure was repeated twice, combined the two decoctions and concentrated to 1 g/ml. Benzbromarone was obtained from Heumann Pharma (Nuremberg, Germany).

### Animal experiments and sample preparation

#### Animals treatments

This study protocol was approved by the Animal Care and Ethics Committee of the First Affiliated Hospital of Zhengzhou University (2022-KY-0627-001, Zhengzhou, China). All animal experiments were carried out in accordance with institutional ethical considerations and guidelines, and were also conducted according to ARRIVE guidelines. Forty male Sprague–Dawley rats (SPF grade, 220 ± 20 g) were obtained from Beijing SPF Laboratory Animal Technology. The animals were maintained in plastic cages with water and food available ad libitum. All animals were housed on a 12 h day-night cycle, and air-conditioner and venting system were used to keep the temperature and humidity at 25 ± 1 °C and 55 ± 5%, respectively. As previously described^21^, 10% fructose water (AMRESCO, USA) was used to induce HUA model. The rats were divided randomly into five groups (n = 8 per group). Among them, the control group (Con) was given normal drinking water, the model group (Mod) was given 10% fructose water, the positive group (Ben) was given 10% fructose water and treated with 20 mg·kg^−1^·d^−1^ benzbromarone, the *C. spicatus* group (CS) was given 10% fructose water and treated with 10.0 g·kg^−1^·d^−1^ *C. spicatus*, and the *C. spicatus* low-dose group (CSL) was given 10% fructose water and treated with 5.0 g·kg^−1^·d^−1^ *C. spicatus*^22^. Similarity, the Con group and the Mod group were gavaged with normal saline (0.9%). All drugs were administered via the stomach once a day.

#### Sample preparation

Serum was taken once a week after fasting 12 h (3000 rpm, 10 min, 4 °C) and the rats were sacrificed after 4 weeks. In addition, at week 4, a portion of the blood sample was placed in a centrifuge tube containing heparin sodium to obtain plasma for metabolomic studies. The serum levels of UA were detected using assay kit (BioSino Biotechnology & Science, Beijing, China), whereas xanthine oxidase (XOD), adenosine deaminase (ADA), BUN and creatinine (Cre) were measured with automatic chemistry analyzer (Chemray 240, Rayto Life and Analytical). Furthermore, the kidneys were excised on ice and fixed in neutral paraformaldehyde for 48 h. Then, formalin-fixed kidneys were cut at a thickness of 4 μm and stained with hematoxylin–eosin (HE). The slides were observed and photographed under a light microscope for the changes of kidney tissue structure.

#### RNA isolation and real-time PCR analysis

Kidney (100 mg) was homogenized with TRIzol Reagent (Invitrogen Life Technologies, Carlsbad, CA, lot number: 15596-026) to extract mRNA. Extracted mRNA was reverse-transcribed following the manufacturer protocol of a HiScript® II Reverse Transcriptase (Vazyme, Nanjing, China, lot number: R211-01). Quantitative real-time polymerase chain reaction (PCR) was carried out using ChamQ Universal SYBR qPCR Master Mix kit (Vazyme, Nanjing, China, lot number: Q711-02), and then fluorescence quantitative analysis was carried out using ABI7500 fluorescence quantitative analyzer produced by ABI Company. Additionally, the primers of genes urate transporter1 (URAT1), glucose transporter 9 (GLUT9), and ATP-binding cassette super-family G member 2 (ABCG2) refer to the relevant sequence information in the article by Zhao et al.^23^.

#### Measurement of protein expression of transporters

Western blotting analysis was performed as our research group previously described. Kidneys of rats were rinsed with PBS, lysed with radioimmunoprecipitation assay buffer and then centrifuged (12,000 rpm, 15 min, 4 °C). 60 μg of lysates were separated by 12% SDS–polyacrylamide gel electrophoresis, and then transferred to polyvinylidene difluoride membrane (Millipore, Bedford, MA, USA). The membranes were blocked with 5% skimmed milk for 1 h at room temperature, and pre-incubated with one of the following antibodies overnight at 4 °C: anti-ABCG2 (1:200 dilution, Abcam, USA, lot number: sc-58222), anti-GLUT9 (1:1000 dilution, Merk, Germany, lot number: ABN407), anti-URAT1 (1:1000 dilution, biorbyt, UK, lot number: orb637320) and mouse-anti-β-actin antibody (1:5000, Proteintech, China, lot number: 66009-1-Ig). After incubation with the secondary antibody, the membrane was washed in TBST for 30 min, and the Bio-RAD exposure instrument was preheated for exposure before color development. Finally, the gray value of the protein band was analyzed by Image J software.

#### Chromatographic and mass spectrometric conditions

The separation was performed on a ACQUITY UPLC HSS T3 C18 column (2.1 mm × 100 mm, 1.8 μm) at 40 °C. The mobile phases consisted of 0.1% formic acid in water (solvent A) and acetonitrile (solvent B). The elution gradient is as follows: 0 min, 95%A; 11 min, 10%A; 12 min, 10%A; 12.1 min, 95%A; 14 min, 95%A, which was delivered at 0.4 mL/min. The volume of injection samples was fixed at 2 μL. The eluent was directly introduced to the mass spectrometer. Quality control samples (QC) are prepared by mixing sample extracts to monitor the reproducibility of analytical samples under the same processing method. In the process of instrument analysis, the quality control sample was inserted into every 8 detection analysis samples to monitor the repeatability of the analysis process. And by tandem mass spectrometry, electrospray ionization: the electrospray capillary voltage was 5500 V in positive ionization mode and 4500 V in negative ionization mode. Both capillary temperature and aux gas heater temperature were 500 °C. The ion source gas I, gas II and curtain gas stepped 55, 60, and 25 psi, respectively.

### Data analysis

Data are represented as the mean ± SD. Statistical analyses were carried out with one-way ANOVA followed by Dunnett’s multiple comparison test to determine levels of significance using SPSS v 20.0 (IBM, Armonk, USA), and *P* < 0.05 was considered significant. Meanwhile, the graphs were made by GraphPad Prism 8.0 software.

By using multiple reaction monitoring of triple-quadrupole mass spectrometry, the metabolites were quantified and the characteristic ions as well as the signal strength were obtained. Then, imported the data into R software and performed multivariate data processing, including principal component analysis (PCA) and orthogonal partial least squares discriminant analysis (OPLS-DA). The variable importance in projection (VIP) of multivariate OPLS-DA model preliminarily filtrated the metabolites from various groups, and variables with VI* P* ≥ 1.0 and fold change ≥ 2 or ≤ 0.5 were selected for further data analysis. Finally, the screened differential metabolites were imported into kyoto encyclopedia of genes and genomes (KEGG) for enrichment analysis of metabolic pathways (www.kegg.jp/kegg/kegg1.html)^24–26^.

## Results

### Effects of *C. spicatus* on serum UA, XOD and ADA levels

**Figure 1:**
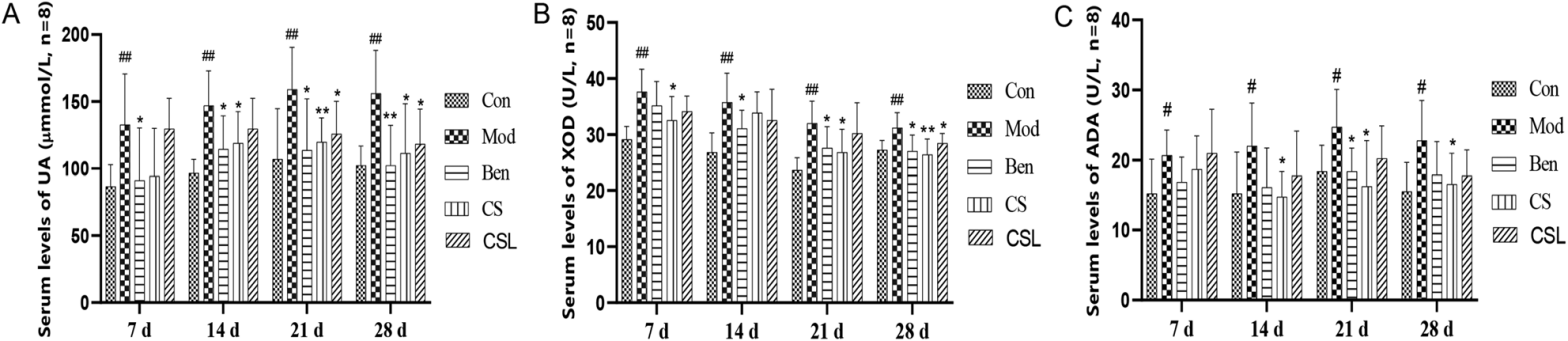
Effects of *C. spicatus* on serum UA levels (**A**), XOD activity (**B**), and ADA activity (**C**) in HUA rats (n = 8). ^#^*P* < 0.05, ^##^*P* < 0.01 versus the Con group; *P < 0.05, **P < 0.01 versus the Mod group. HUA, Hyperuricemia; UA, Uric acid; XOD, Xanthine oxidase; ADA, Adenosine deaminase.

The serum levels of UA were measured as a characteristic HUA index. As shown in Fig. 1A, 10% fructose-drinking caused a significant increase in serum UA levels (*P* < 0.01) within 7–28 d, indicating that the HUA model was successfully constructed. Conversely, the serum UA levels of the Ben group were obviously lower than those of the Mod group (*P* < 0.05 or *P* < 0.01) during the entire study, and the CS group showed significantly reduced serum UA levels (*P* < 0.05 or *P* < 0.01) during 14–28 d. In parallel, the serum UA levels of the CSL group were significantly reduced in 21–28 d (*P* < 0.05), but showed no significant difference during the first 7–14 d (*P* > 0.05). As key enzymes in purine metabolism, XOD and ADA are essential for the development of HUA^27,28^. Figure 1B illustrates that, compared with the Con group, serum XOD levels in the Mod group were significantly increased during 7–28 d (*P* < 0.01). On the 14–28 d of the experiment, the serum XOD levels of the Ben group were significantly lower than those of Mod group (*P* < 0.05). Of note, different doses of *C. spicatus* could attenuate the serum XOD levels in HUA rats. Particularly, compared with the Mod group, the serum XOD levels of the CS group were notably reduced (*P* < 0.05 or *P* < 0.01) on days 7, 21 and 28, and the CSL group were significantly decreased on day 28 (*P* < 0.05). Furthermore, it could be seen from Fig. 1C that the serum ADA levels in the Mod group were significantly higher than those in the Con group (*P* < 0.05). However, different doses of *C. spicatus* could reduce ADA levels in HUA rats to different degrees.

### Effects of *C. spicatus* on kidney damage

**Figure 2:**
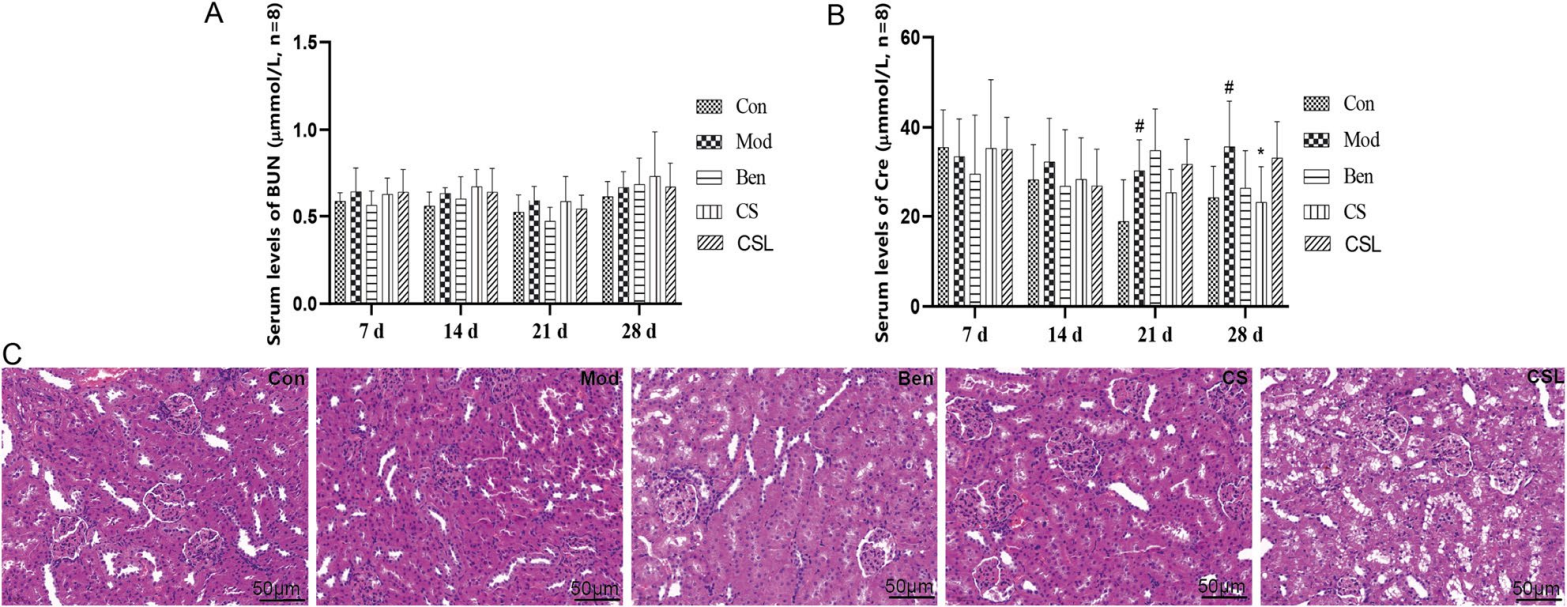
Effects of *C. spicatus* on kidney damage. Serum levels of BUN (**A**); Serum levels of Cre (**B**); Typical histopathological section photographs of the kidney for HE analysis (**C**). (magnification 400 × ; scale bar = 50 μm). ^#^*P* < 0.05 versus the Con group; *P < 0.05 versus the Mod group. BUN, Blood urea nitrogen; Cre, Creatinine.

Furthermore, the serum levels of BUNand Cre were measured as indicators of kidney damage. As shown in Fig. 2A, there was no significant difference (*P* > 0.05) in the BUN levels among groups during the administration period. Surprisingly, Fig. 2B showed that the serum Cre levels in the Mod group were significantly higher than those in the Con group during 21–28 d (*P* < 0.05), whereas CS treatment significantly decreased Cre levels at 28 d (*P* < 0.05).

Alongside, as shown in Fig. 2C, HE-stained kidney sections from the Con group revealed normal histology with glomeruli and tubules. Compared with the Con group, stained sections from the Mod group showed that the cytoplasm was lightly stained, the renal tubules were obviously dilated, and some nuclei were disorganized. Stained sections from the Ben group showed an obvious improvement of renal tubular dilation. Compared with the Mod group, improved morphologic structures of glomeruli and renal tubules were observed in the CS and CSL groups, suggesting that *C. spicatus* could improve kidney damage.

### Effects of *C. spicatus* on UA transporter expression

**Figure 3:**
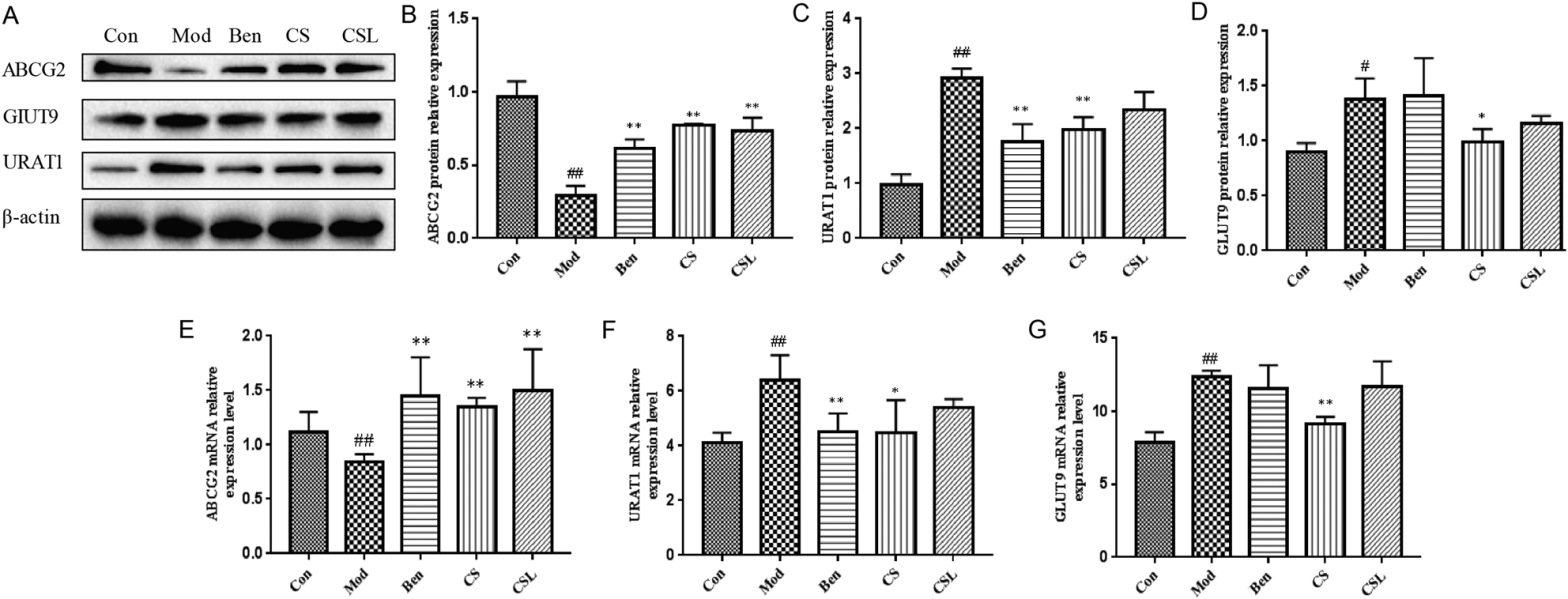
Effects of *C. spicatus* on UA transporter expression in the kidney. GLUT9, URAT1 and ABCG2 protein levels in each group (**A**–**D**); GLUT9, URAT1 and ABCG2 mRNA levels in each group (**E**–**G**). ^#^*P* < 0.05, ^##^*P* < 0.01 versus the Con group, *P < 0.05, **P < 0.01 versus the Mod group. URAT1, Urate transporter1; GLUT9, Glucose transporter 9; ABCG2, ATP-binding cassette super-family G member 2.

Effects of *C. spicatus* on the expression of UA transporters in HUA rats were shown in Fig. 3A–D. Compared with the Con group, the expression of ABCG2 was significantly decreased (*P* < 0.01), while the expression of URAT1 and GLUT9 was significantly increased in the Mod group (*P* < 0.05 or *P* < 0.01). Notably, the protein expression of renal URAT1 and GLUT9 was remarkably reduced in the CS group (*P* < 0.05 or *P* < 0.01). Similarity, the BEN group showed a notable reduction in the expression of renal URAT1 (*P* < 0.01). Nevertheless, URAT1 and GLUT9 protein expression had no changes in the CSL group (*P* > 0.05). On the other hand, it was worthy to note that the expression of ABCG2 in the Ben, CS and CSL groups was significantly increased when compared with HUA rats (*P* < 0.01) (Fig. 3B–D).

In accordance with protein levels, the mRNA levels of GLUT9 and URAT1 were markedly increased in HUA rats compared with the Con group (*P* < 0.01) (Fig. 3F,G). Furthermore, CS treatment could significantly decrease the mRNA levels of GLUT9 and URAT1 in HUA rats (*P* < 0.05 or *P* < 0.01), whereas there were no notable changes in the CSL group (*P* > 0.05). Moreover, the mRNA levels of ABCG2 in HUA rats were significantly decreased compared with those in the Con group (*P* < 0.01), and the Ben, CS and CSL groups showed notably increased the mRNA levels of ABCG2 compared with the Mod group (*P* < 0.01) (Fig. 3E).

### Multivariate statistical analysis of *C. spicatus* on HUA

**Figure 4:**
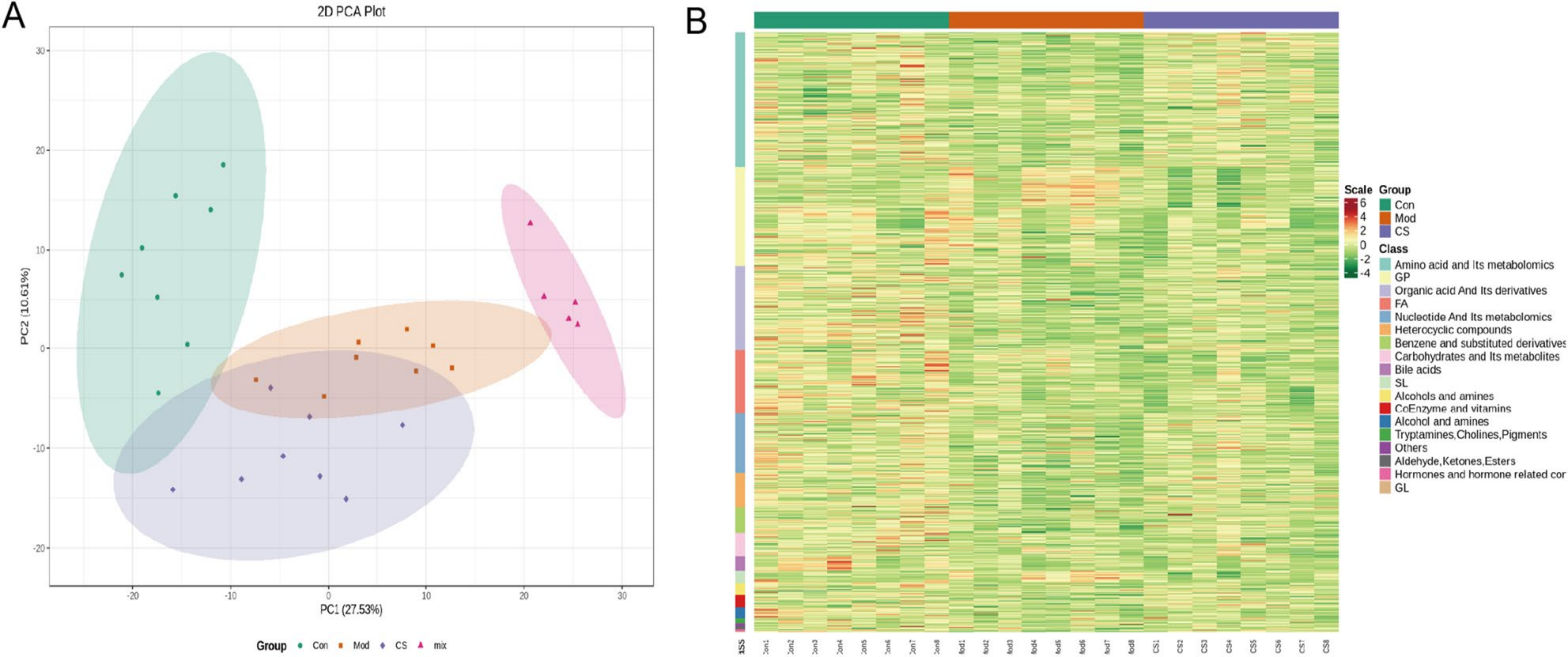
Multivariate statistical analysis of *C. spicatus* on HUA. (**A**) PCA score plot of all analyzed samples (Con: green round, Mod: orange square, CS: light purple diamond, and MIX: rose red triangle). (**B**) Cluster analysis (red indicating higher metabolite concentrations and green indicating lower metabolite concentrations). HUA, Hyperuricemia; PCA, Principal component analysis.

The CS group exhibited a better UA-lowering advantage compare to the CSL group. Therefore, to determine the effects of *C. spicatus* on metabolic pathways, we analyzed serum metabolites in the Con, Mod, and CS groups. Firstly, we performed data evaluation on each sample. The total ion current (TIC) plots of the mixed samples were shown in Fig. S1. The TIC overlap between samples is high, and the retention time and peak intensity are consistent, indicating high stability and reliability of UPLC analysis (Fig. S2). Noteworthy, PCA analysis (Fig. 4A) showed that there was an obvious separation between the Con and Mod groups, while the CS group partially overlapped with the Mod group, but deviated from the Con group, which preliminarily indicated that *C. spicatus* improved the metabolic disorder after modeling. Furthermore, cluster analysis (Fig. 4B) showed that the metabolites mainly included amino acid and its metabolomics, organic acid and its derivatives, as well as benzene and substituted derivatives.

### Identification of potential biomarkers for *C. spicatus* treatment

**Figure 5:**
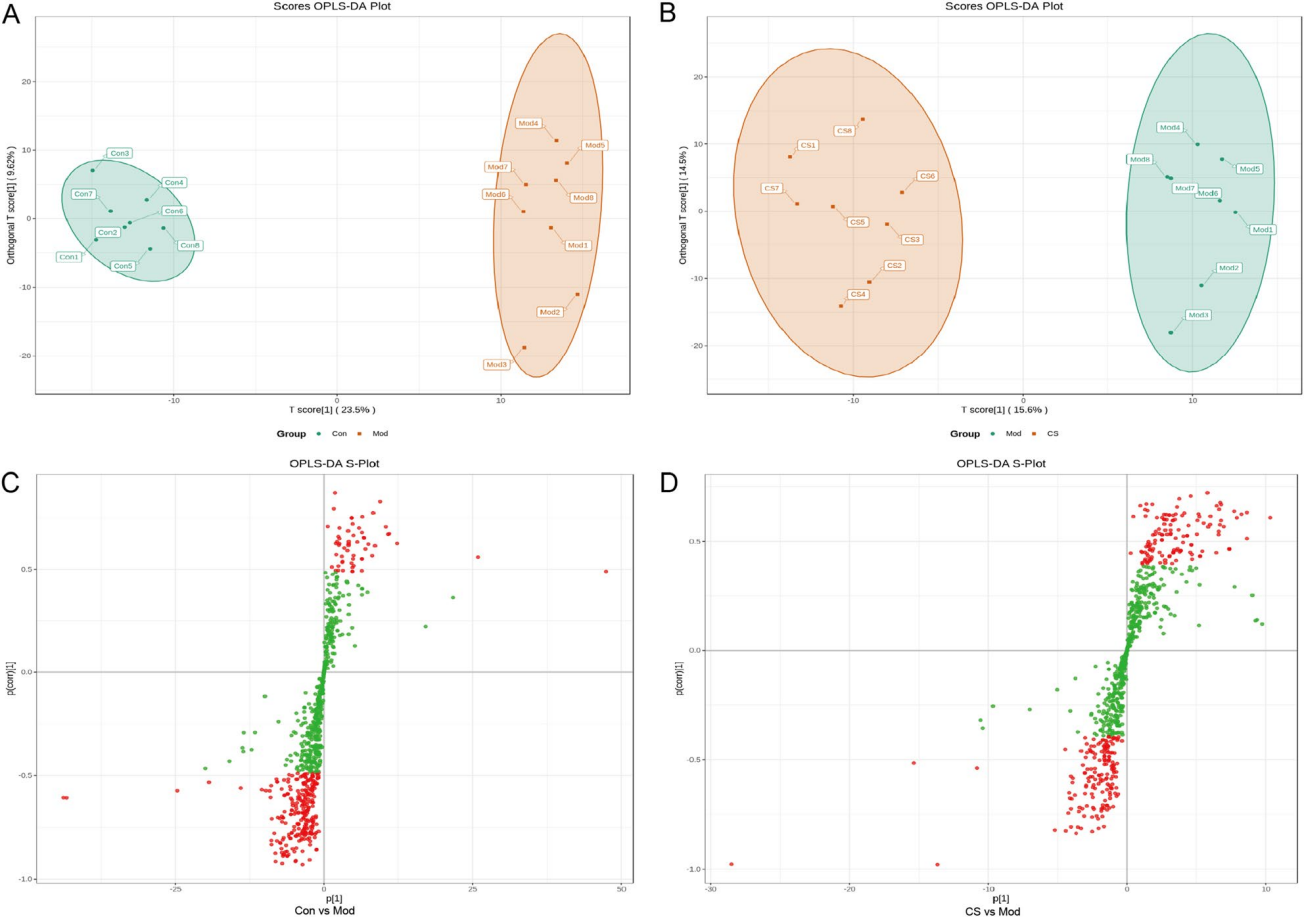
Establishment and validation of OPLS-DA model. OPLS-DA analysis between the Con group and the Mod group (**A**); OPLS-DA analysis between the Mod group and the CS group (**B**); S-plot analysis for the Con and Mod groups (**C**); S-plot analysis for the Mod and CS groups (**D**). OPLS-DA, Orthogonal partial least squares discriminant analysis.

OPLS-DA was then applied to further identify potential biomarkers showing prominent concentration changes. As seen from Fig. 5A,B, the OPLS-DA results showed that the pairwise comparisons of the Con, Mod, and CS groups had obvious separation trends. S-plot analysis (Fig. 5C,D) further showed that the distribution of differential metabolites between groups (VIP ≥ 1.0 and fold change ≥ 2 or ≤ 0.5 were considered as the potential discriminant variables and identified as candidate biomarkers). Of interest, a total of 13 of the 67 biomarkers were simultaneously altered with opposing trends in Mod and CS groups (Table 1 and Fig. S3). Particularly, compared with the Mod group, the levels of 1-Palmitoyl-2-Arachidonoyl-sn-glycero-3-PE, PE (16:1e/14-HDoHE), PC (18:2(9Z,12Z)/P-18:1(11Z)), PC (18:1(9Z)/P-18:1(11Z)), PC (16:0/P-16:0), PC (18:2(9Z,12Z)/15:0), SM (d18:1/22:0), PE (22:6(4Z,7Z,10Z,13Z,16Z,19Z)/P-16:0), N-cis-15-Tetracosenoyl-C18-sphingosine, PC (O-16:0/18:1), N-nervonoyl-D-erythro-sphingosylphosphorylcholine and 1,2-Dioleoyl-sn-glycero-3-phosphocholine were significantly down-regulated in the CS group. Nevertheless, the levels of Tyr-Leu were markedly up-regulated in the CS group compared with the Mod group.

**Table 1:** The identification of potential biomarkers based on metabolomics.

Index	Formula	Identification	Con versus Mod	Mod versus CS
MADN0469	C41H74NO8P	Palmitoyl-2-Arachidonoyl-sn-glycero-3-PE	Up	Down
MADN0482	C43H74NO8P	PE(16:1e/14-HDoHE)	Up	Down
MADP0435	C44H82NO7P	PC(18:2(9Z,12Z)/P-18:1(11Z))	Up	Down
MADP0437	C44H84NO7P	PC(18:1(9Z)/P-18:1(11Z))	Up	Down
MADP0439	C40H80NO7P	PC(16:0/P-16:0)	Up	Down
MADP0450	C41H78NO8P	PC(18:2(9Z,12Z)/15:0)	Up	Down
MADP0452	C45H91N2O6P	SM(d18:1/22:0)	Up	Down
MADP0460	C43H74NO7P	PE(22:6(4Z,7Z,10Z,13Z,16Z,19Z)/P-16:0)	Up	Down
MADP0466	C42H81NO3	N-cis-15-Tetracosenoyl-C18-sphingosine	Up	Down
MADP0467	C42H84NO7P	PC(O-16:0/18:1)	Up	Down
MADP0471	C47H93N2O6P	N-nervonoyl-D-erythro-sphingosylphosphorylcholine	Up	Down
MADP0475	C44H84NO8P	1,2-Dioleoyl-sn-glycero-3-phosphocholine	Up	Down
MEDP1894	C15H22N2O4	Tyr-Leu	Down	Up

### Pathway analysis of *C. spicatus* on HUA

**Figure 6:**
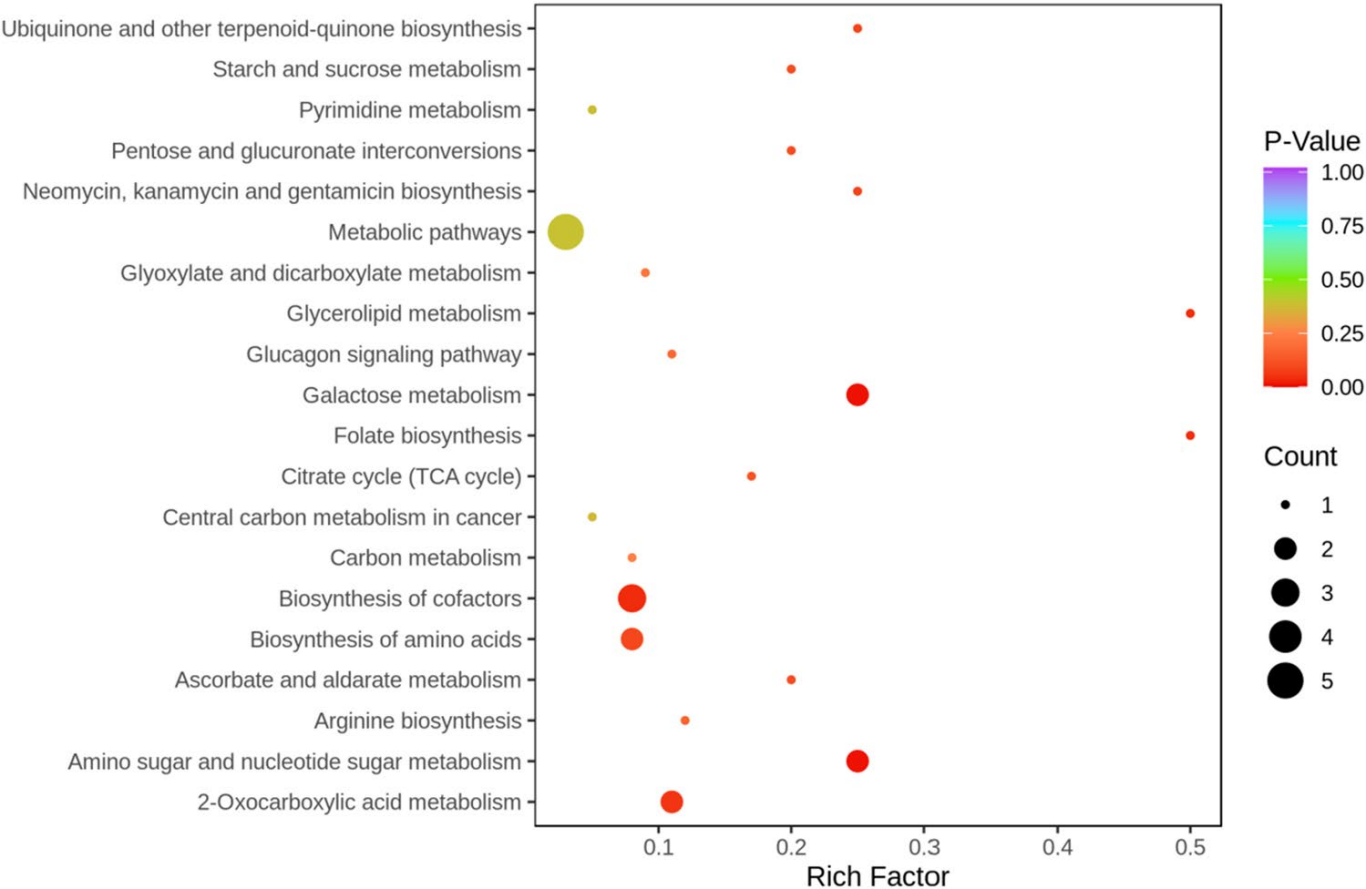
KEGG pathway enrichment results in the treatment of HUA with *C. spicatus*. HUA, Hyperuricemia; KEGG, Kyoto encyclopedia of genes and genomes.

Subsequently, all candidate biomarkers were analyzed for KEGG pathway enrichment. As a result, metabolic pathway enrichment analysis revealed that 20 pathways contributed to HUA, including arginine biosynthesis, biosynthesis of amino acids, pyrimidine metabolism, and glucagon signaling pathway (Fig. 6).

## Discussion

Growing evidence has indicated that excessive consumption of fructose is involved in metabolic syndrome, chronic kidney disease and HUA^29,30^. Indeed, the metabolism of fructose stimulates AMP deaminase, which in turn increases the rate of purine degradation^31^. Furthermore, it has been reported that long-term intake of fructose inhibits the excretion of UA from the kidney, resulting in increased serum UA levels^32^. Nonetheless, the effect of short-term intake of fructose on kidney excretion of UA is not completely understood. In the present study, we found that the serum UA of the Mod group could maintain a significantly elevated level throughout the study (Fig. 1A). This result is also in line with the current findings by Wang^33^. Additionally, renal dysfunction in HUA rats was documented (Fig. 2A,B). There were no significant BUN changes between the Con and the Mod groups. However, compared with the Con group, serum Cre levels in the Mod were significantly increased on days 21–28. On the other hand, HE-stained kidney sections showed that the renal cytoplasm was lightly stained, the renal tubules were significantly expanded, and some nuclei were disorganized after drinking fructose water for 28 days. Overall, these findings suggest that short-term fructose-induced HUA may be associated with renal impairment.

Recently, the genome-wide association studies and some large meta-analyses have revealed that the UA transporters are closely related to HUA^34,35^. In the kidney, the UA transporters were mainly divided into two categories: UA reabsorption transporters and UA excretion transporters, which is mediated mainly by URAT1, GLUT9 and ABCG2^36,37^. URAT1 is a 12-transmembrane domain protein, which regulates tubular reabsorption of UA through ion-exchange mechanism with organic anions on the luminal membrane of epithelial cells in the proximal convoluted tubule of the kidney^38,39^. In the kidney proximal tubule, GLUT9 transports UA into the serum through the basolateral membrane, and it is a high-capacity UA transporter that plays an important role in the homeostasis of serum UA levels^40^. As an ATP-binding cassette transporter 2, ABCG2 plays a crucial role in renal UA excretion, and increasing evidence shows that ABCG2 dysfunction is a risk factor for HUA^41^. As mentioned above, a series of transporters regulate the renal excretion of UA. In this study, the expression of URAT1 and GLUT9 was significantly increased, while the expression of ABCG2 was significantly decreased in HUA rats. In addition, the mRNA levels were consistent with the above protein levels. Consistent with the results, Yang et al.^42^ also found that fructose drinking could cause significant changes in the above-mentioned UA transporters. Previous study had indicated that 12 compounds isolated from *C. spicatus* can promote the excretion of UA at 10 μg/mL^13^. In the present study, compared with the Mod group, the protein expression of URAT1 and GLUT9 in the CS group was significantly reduced, whereas ABCG2 was significantly increased in the CS group. All the results suggested that *C. spicatus* significantly decreased serum UA levels in HUA rats possibly by regulating the expression URAT1, GLUT9 and ABCG2.

Metabolomics provides important clues for elucidating the pathophysiological mechanisms. With the advent and evolution of metabolomics’ technologies, the mechanisms studies related to HUA have developed rapidly. For example, Chen et al.^43^ found that arginine biosynthesis, galactose metabolism, TCA cycle and pyrimidine metabolism were the main metabolic pathways for Tongfengxiaofang in the treatment of HUA by metabolomics. Huang et al.^44^ used metabolomic approach to study the potential mechanism by which Er Miao Wan reduced UA, and found that these metabolites were mainly associated with glycerolipid metabolism, primary bile acid metabolism and purine metabolism. In the study, a total of 13 biomarkers were significant altered and the trend was reversed after *C. spicatus* treatment. Interestingly, Murota et al. found that oral administration of peptide containing Tyr-Leu had anti-HUA activity^45^. The present study found that Tyr-Leu was significantly increased in the CS group compared with the Mod group, suggesting that *C. spicatus* may exert UA-lowering effects by increasing Tyr-Leu. Further metabolic pathway analysis revealed that the variations of these metabolites were mainly associated with arginine biosynthesis, pyrimidine metabolism, pentose and glucuronate interconversions, glycerolipid metabolism, glucagon signaling pathway, galactose metabolism, folate biosynthesis, citrate cycle (TCA cycle), and biosynthesis of amino acids. Notably, previous studies have shown that high levels of UA stress cardiomyocytes by accelerating arginine metabolism through the up-regulation of ornithine decarboxylase, which may be an important factor in the development of HUA-associated cardiac disease^46^. Surprisingly, purine metabolism, as the terminal pathway for UA production, was not screened in this experiment, suggesting that *C. spicatus* may exert its UA-lowering effects through other mechanisms.

## Conclusion

The present study demonstrated the anti-HUA property of *C. spicatus* in a classical rat model of HUA and highlighted its potential mechanisms by an integrated approach of molecular biology analysis and metabolomics. In conclusion, *C. spicatus* protects rats from HUA via improving renal function, regulating UA transporters (URAT1, GLUT9, and ABCG2) and remodeling metabolic disorders. This research will lay a theoretical foundation for the use of *C. spicatus* as a UA-lowering drug. However, the molecular mechanism needs to be further explained and enriched.

### Supplementary Information

Supplementary Figures.

## Data Availability

The datasets generated during and/or analysed during the current study are available from the corresponding author on reasonable request.

## Abbreviations

HUA: Hyperuricemia
UA: Uric acid
*C. spicatus*: *Clerodendranthus spicatus*
URAT1: Urate transporter1
GLUT9: Glucose transporter 9
ABCG2: ATP-binding cassette super-family G member 2
XOD: Xanthine oxidase
ADA: Adenosine deaminase
BUN: Urea nitrogen
Cre: Creatinine
HE: Hematoxylin–eosin
PCR: Polymerase chain reaction
QC: Quality control samples
PCA: Principal component analysis
OPLS-DA: Orthogonal partial least squares discriminant analysis
VIP: Variable importance in projection
TIC: Total ion current
