# Supplementary material for: Anti-hyperuricemia effect of Clerodendranthus spicatus: a molecular biology study combined with metabolomics
Source: Sci Rep. 2024 Jul 4;14:15449. doi: 10.1038/s41598-024-66454-7 (PMC11224374; doi:10.1038/s41598-024-66454-7)
Supplement: Supplementary file 1 — Supplementary Figures. [file 41598_2024_66454_MOESM1_ESM.pptx]

## Slide 1
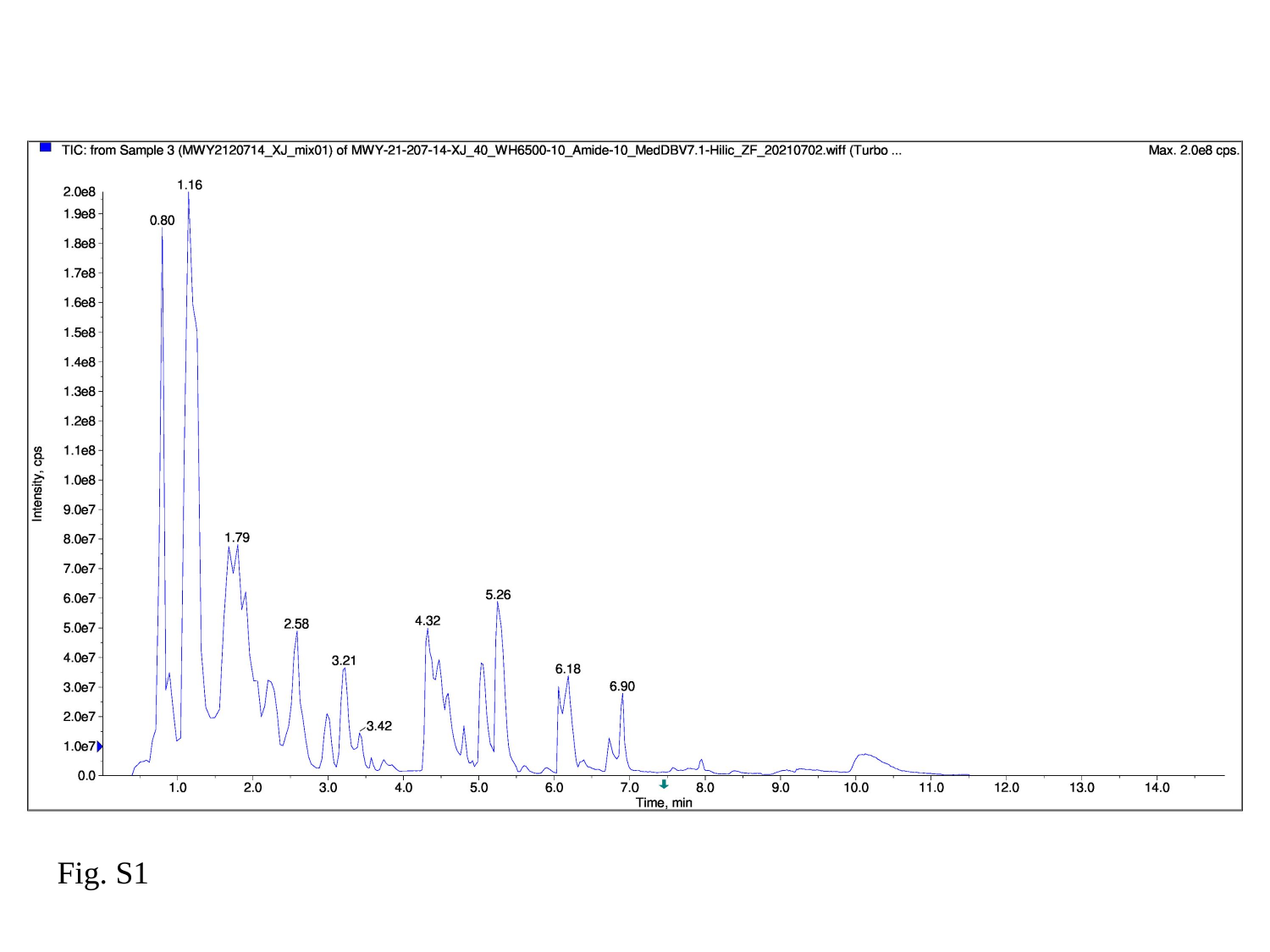

Fig. S1

## Slide 2
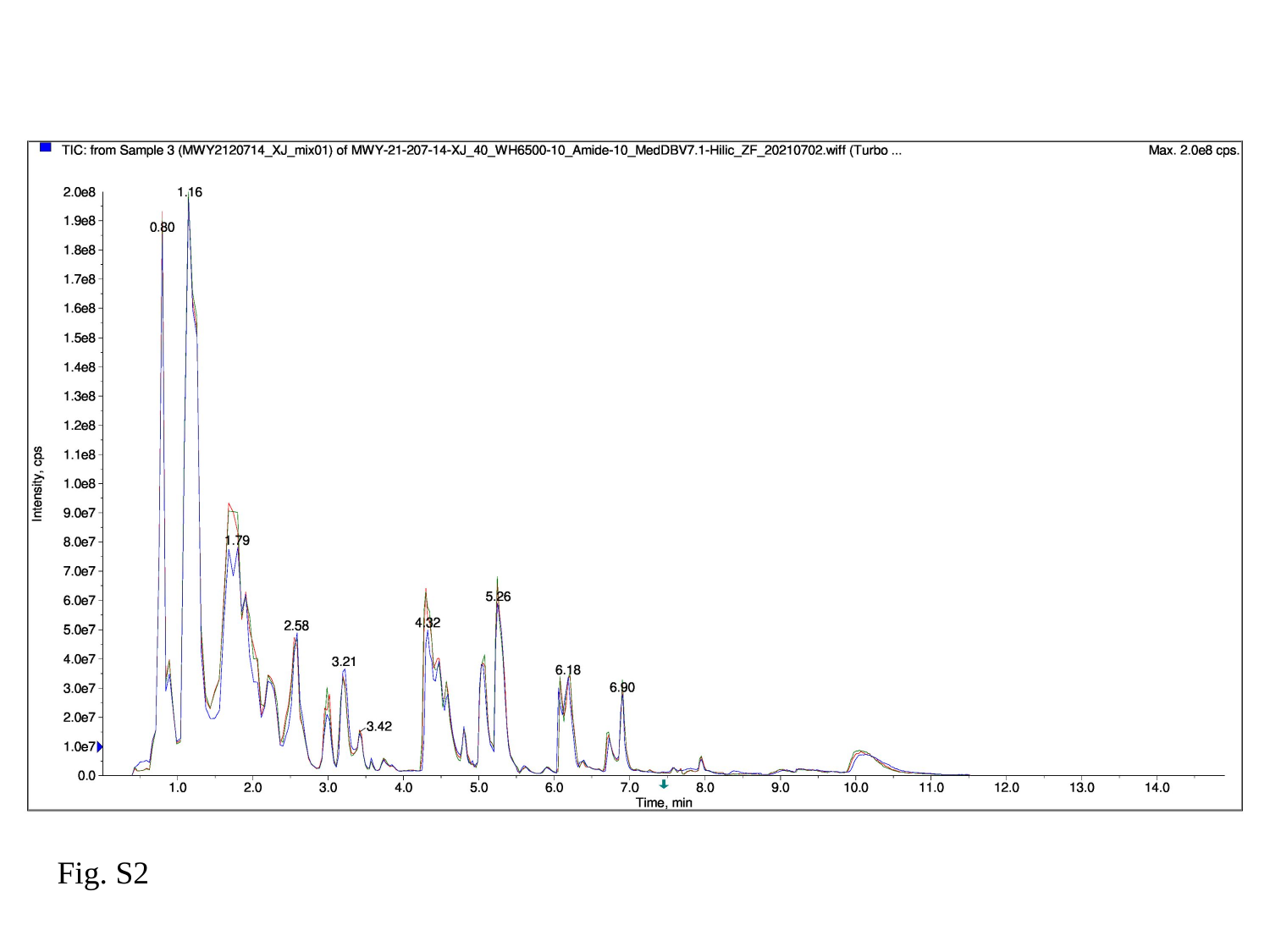

Fig. S2

## Slide 3
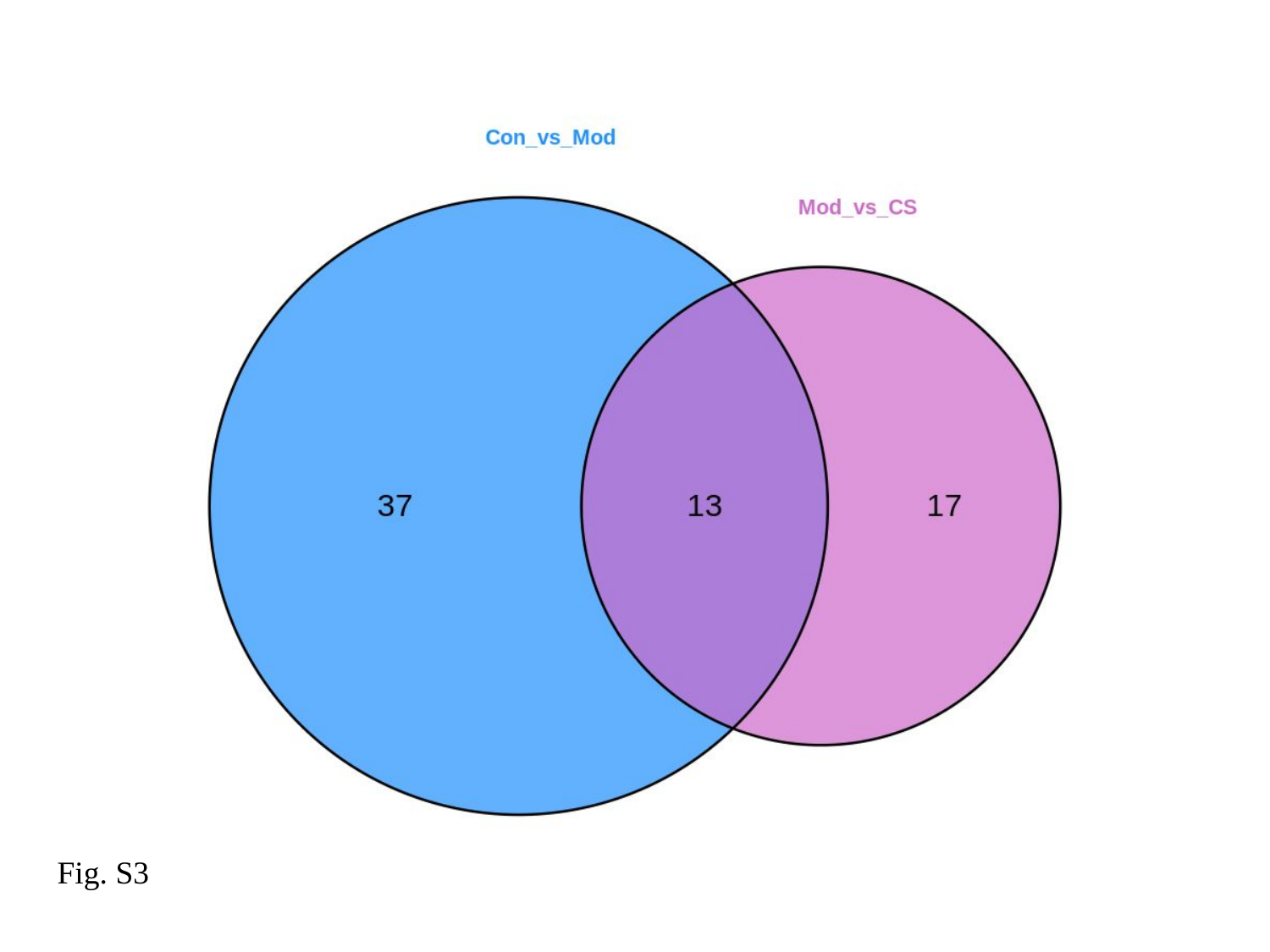

Fig. S3

## Slide 4
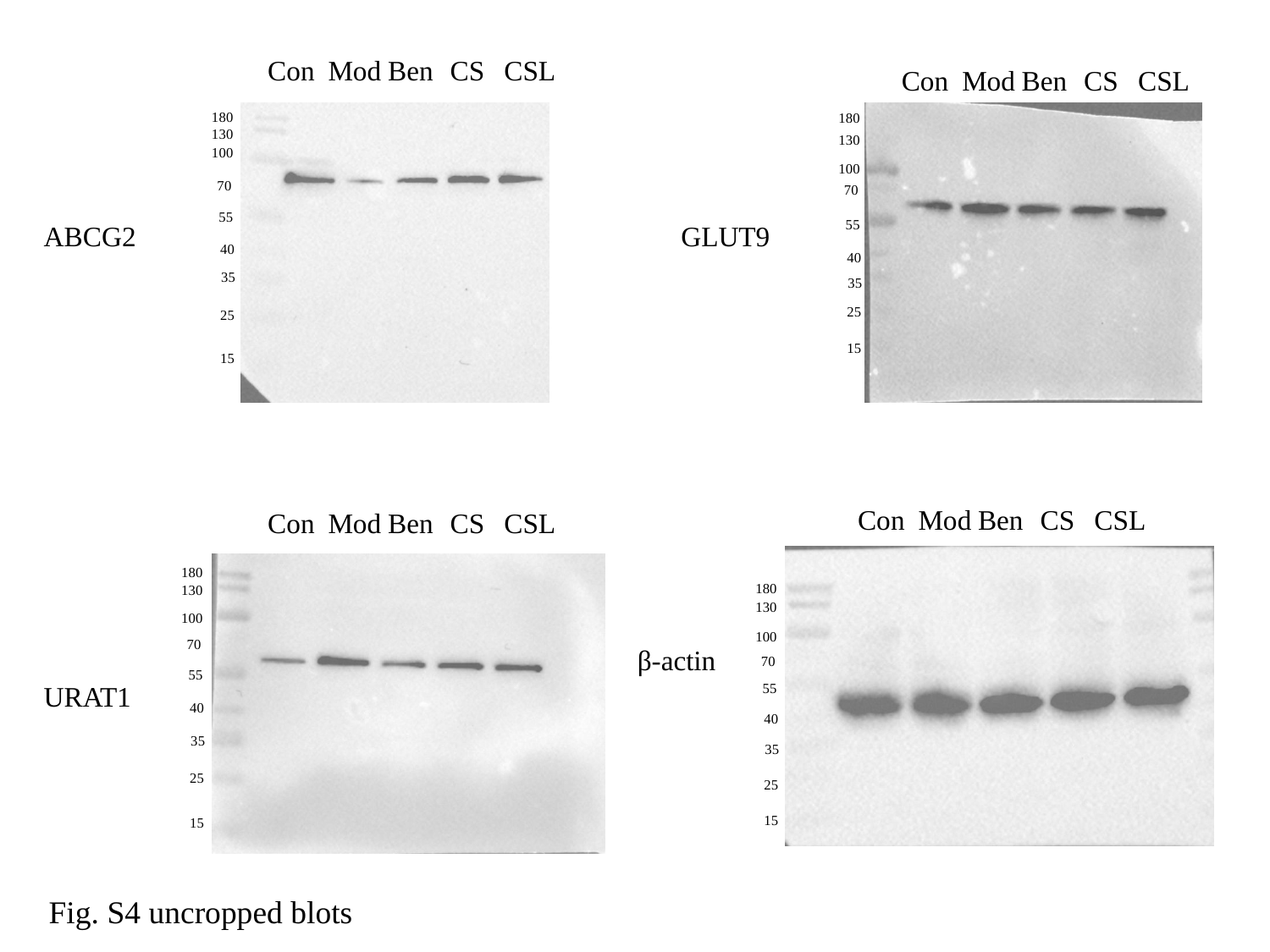

Con
Mod
Ben
CS
CSL
Con
Mod
Ben
CS
CSL
180
180
130
130
100
100
70
70
55
55
ABCG2
GLUT9
40
40
35
35
25
25
15
15
Con
Mod
Ben
CS
CSL
Con
Mod
Ben
CS
CSL
180
180
130
130
100
100
70
β-actin
70
55
URAT1
55
40
40
35
35
25
25
15
15
Fig. S4 uncropped blots
